# Knowledge Hiding in Emergency Ambulance Healthcare Settings: Its Mediating Role in the Relationship between Organizational Support and Affective Commitment and Organizational Citizenship Behaviours

**DOI:** 10.3390/nursrep11040088

**Published:** 2021-11-29

**Authors:** Lucia Ratiu, Sabina R. Trif, Nicoleta Meslec

**Affiliations:** 1 Psychology Department, Babeş-Bolyai University, 400084 Cluj-Napoca, Romania; sabinatrif@psychology.ro; 2 Department of Organization Studies, Tilburg University, 5037 AB Tilburg, The Netherlands; M.N.Meslec@tilburguniversity.edu

**Keywords:** knowledge hiding, perceived organizational support, affective commitment, organizational citizenship behaviors, turnover intentions

## Abstract

Knowledge hiding—an intentional attempt to withhold or conceal knowledge from others—has been reported by recent studies to be a negative phenomenon in the workplace. Considering the importance of knowledge for organizational performance, this study intends to advance understanding by investigating the mediating role of knowledge hiding on the relationship between perceived organizational support and affective commitment as predictors and organizational citizenship behaviors and turnover intentions as outcomes. Using a cross-sectional design, the study was conducted in emergency ambulance healthcare settings on 305 medical or paramedical professionals. As indicated by structural equation modeling results, perceived organizational support and affective commitment positively predicted organizational citizenship behaviors but negatively predicted turnover intentions. Also, knowledge hiding was negatively associated with perceived organizational support, affective commitment, and organizational citizenship behaviors and positively with turnover intentions. Moreover, knowledge hiding mediated the relationship between perceived organizational support and affective commitment as predictors and organizational citizenship behaviors, respectively turnover intentions, as dependent variables.

## 1. Introduction

Knowledge hiding has gained increasing attention in recent years in the field of organizational behavior, especially concerning its potential influence on organizational performance [1,2,3,4,5,6,7]. Knowledge sharing, knowledge hiding, and knowledge manipulation are the main streams of literature developed to explain individuals’ tactical choices involving knowledge [2,8]. Thus far, the focus in the knowledge management literature is typically on knowledge sharing and less on the other processes involving knowledge (e.g., knowledge hiding).

Unfortunately, knowledge hiding among employees frequently occurs in the workplace because of the competitive environment having the potential to affect working relations and so knowledge hiding can become a pressing issue [9]. Given its growing importance, scholars have examined various personal and contextual contingents of knowledge hiding in organizations [10,11,12,13,14,15]. Nevertheless, knowledge hiding represents a new topic in knowledge management research, which has not been thoroughly researched. We largely accept that knowledge hiding is not beneficial for the organizations at all levels, including individual and team levels, but we need to deepen the understanding of specific consequences within organizations [16].

Beyond the negative influence knowledge hiding could have, the antecedents of such behavior could be relevant for the organizations. How bad is it to hide knowledge? What are the antecedents of the knowledge hiding behaviors? How can we explain the relationships knowledge hiding has with other behaviors? What organizational components might be relevant to mitigate its negative influence? Importantly, barriers to exchange vary depending on the locations of the knowledge source and seeker and it is relevant to identify the factors that facilitate or impede the knowledge exchange [17]. While factors related to knowledge sharing have been studied intensely [8], those that influence knowledge hiding have been overlooked.

Given the paucity of research in the field of knowledge hiding, we aim to study in this paper the contingent factors of knowledge hiding. What’s wrong with lacking and avoiding transparency at work? What predicts knowledge hiding in organizations? Given the difficulties related to convincing employees to share their knowledge, organizations should understand the dynamics that may enable knowledge sharing within its boundaries.

Although the emergent literature on knowledge hiding is promising [2,10], specific situations remain open to investigation [12]. More precisely, it is still unclear how knowledge hiding may relate to organizational citizenship behaviors and turnover intentions. Thus, we aim to explore the antecedents and consequences of knowledge hiding and its explanatory role. We add to the literature by disentangling the effect of both perceived organizational support and affective commitment on knowledge hiding, alongside exploring the role of knowledge hiding as an explanatory mechanism between both perceived organizational support and affective commitment on one hand, and citizenship behaviors and turnover intentions on the other hand.

In the following section, we will present the literature relevant to the proposed relationships. We will further present the methodological approach to testing our hypotheses, including details about the sample, procedure, and materials used. We will then present the results in a dedicated section, where we test each hypothesis and discuss whether they were supported by our data. In the end, we will have a discussion section, where we will integrate the results in the literature and discuss implications and limitations.

### 1.1. Literature Review

#### 1.1.1. Defining Knowledge Hiding

Knowledge hiding refers to a dyadic relationship between an individual requesting knowledge from another, who, in response, withholds that knowledge [12]. Knowledge hiding “is not simply the absence or the opposite of sharing, but they are conceptually distinct constructs; rather, knowledge hiding is the intentional attempt to withhold or conceal knowledge that has been requested by another individual” [12]. For example, one employee may inquire to his supervisor about a particular issue. The supervisor may choose to only partially disclose knowledge or may simply decline to share any piece of knowledge. Similar situations may arise in vertical or hierarchical relationships across the organization.

Connelly and colleagues (2012) stress that knowledge hiding is different from a lack of knowledge sharing because knowledge hiding adds the intentional withholding component, in addition to simply omitting information.

Knowledge hiding includes three related behaviors: playing dumb, evasive hiding, and rationalized hiding [12]. In fact, in organizations, knowledge hiding is triggered by knowledge requests from others, thus emerging a deception process of varying levels. This intentional process of knowledge hiding hinders positive organizational outcomes [12]. Each of these types of knowledge hiding behaviors can be expected to have both different antecedents and different relationships with work outcomes.

#### 1.1.2. Perceived Organizational Support and Knowledge Hiding

There are some organizational elements that influence the probability of knowledge hiding arising, such as the way performance is appraised [18]—if the organization is focused on individual performance, it will lead to more knowledge hiding behaviors compared to a group appraisal system. If the organization promotes collective performance, then individuals will not be so inclined to knowledge hiding. Besides that, norms such as secrecy play an important role in promoting knowledge hiding [19].

Another important element that may influence the emergence of knowledge hiding is organizational support. Organizational support represents the global beliefs of employees about the extent to which themselves and their values are valued by the organization [20].

According to social identity theory [21], an important part of an individual’s identity is based on his social context. When an organization shows high levels of care of support, individuals will tend to integrate the organization they belong to into their own identity [22]. On the contrary, an organization that doesn’t show support and appreciation to its employees will determine the individuals to include other groups in their identity, leaving their organization behind. In the same vein, Eisenberger and the collaborators (2001) [23] show that when perceived organizations support is high, employees will develop a greater attachment to the organization, and the lack of support will turn the employees away.

In turn, identification and attachment make them deploy more effort into their work in order to attain the organization’s goals [22]. Given that organizational goals take the lead, leaving individual goals behind, employees may not have a reason to engage in knowledge hiding. According to the motivational dilemma framework [24], the opposite is also present—not identifying with the organization and not showing attachment towards may raise the salience of individual goals, promoting knowledge hiding. As such, it is possible that the process of information becoming public will create discomfort, stemming from mixed motives or misaligned goals [25]. Schultze and Stabell (2004) [26] show that knowledge hiding stems from a mismatch between self-goals and team or organizational goals.

In addition, based on the social exchange theory [27], at work, individuals engage in social exchange processes that generate different obligations. Thus, the way a person acts is dependent on another individual’s actions. By the means of these exchanges, the parts involved create a relationship based on trust and loyalty. The development of such relationships is conditioned by some rules and norms—reciprocity being a very important one [28]. When an organization doesn’t offer support and doesn’t provide for its employees, then, by the means of reciprocity, they will be motivated to act in a selfish manner, which prompts the emergence of knowledge hiding.

Thus, we propose the following hypothesis:

**Hypothesis** **1** **(H1).** *The level of perceived organizational support is negatively related to the levels of knowledge hiding in an organization*.

#### 1.1.3. Knowledge Hiding as a Mediator between Perceived Organizational Support and Organizational Citizenship Behaviors

Although there is some research on reasons for engaging in knowledge hiding behaviors (expressed, for example, by evasive hiding, rationalized hiding, and playing dumb), less is known about their consequences [16]. As knowledge hiding is an intentional attempt to conceal or to withhold knowledge that others have requested, it may represent a threat to beneficial outcomes [2,12]. Like other negative work behaviors, it is rarely self-reported, having unanticipated consequences organizations and managers need to address [10].

One important outcome variable, which weighs heavily in the performance of an organization is citizenship behaviors [29,30,31]. Citizenship performance refers to extra behaviors, beyond employees’ formal requirements that actively benefit the organization (e.g., helping co-workers) [32].

A predictor for the emergence of organizational citizenship behaviors could be perceived organizational support. When employees feel valued and see the organization as supportive, they will tend to reciprocate with citizenship behaviors oriented towards the organization [33]. Moreover, variables related to employee morale, such as support, are the most predictive for citizenship behaviors [31]. Piercy and colleagues (2006) [34] also show that, next to control behaviors from leadership, perceived organizational control is an important antecedent of these behaviors, motivating employees to do well for their organization.

As previously argued, based on the social identity theory [21] and on the social exchange theory [27], perceived organizational support negatively predicts knowledge hiding. In turn, knowledge hiding may negatively predict organizational citizenship behaviors, as knowledge hiding may be seen as a way of avoiding unpleasant consequences of knowledge sharing [35]. For instance, experts may want to hide information to keep their status high within the organization or to avoid a time-consuming process. Thus, when the perceived organizational support is high, the knowledge hiding levels will be low, which will raise the probability of citizenship behaviors, given that individuals do not have to protect themselves from the perils of opening up.

Therefore, we propose the following hypothesis:

**Hypothesis** **2** **(H2).** *Knowledge hiding mediates the relationship between perceived organizational support and organizational citizenship behaviours*.

#### 1.1.4. Knowledge Hiding as a Mediator between Perceived Organizational Behaviours and Turnover

Increasingly complex work environments and tremendous cost constraints have challenged organizations to find unique ways to attract, motivate, and retain valued employees [36,37].

Turnover intentions refer to “employees’ voluntary severance of employment ties” [38]. Employee turnover has been a vital issue for management and applied psychology and research studies, as well as meta-analytic tests, show negative relationships between turnover rates and organizational performance [39,40].

Perceived organizational support may act as a buffer for turnover intentions. Based on organizational support theory [20], employees who see their organization as supportive will develop higher levels of organizational identification, which, in turn, will lower the probability of employees wanting to leave the organization [33]. Also, the social exchange theory [27] informs that for a working relationship to be maintained, it is important that both parties perceive the relationship as valuable. In the case of organizational support, employees will feel they receive recognition and that their needs are satisfied, which will lower the chance to leave the organization. Conversely, if they don’t feel appreciated and if they don’t perceive the relationship as valuable, they will manifest turnover intentions.

But this relationship may also be explained by the means of knowledge hiding in organizations, in the sense that, as previously argued, when an organization doesn’t offer the support expected by its employees, they will engage in knowledge hiding. This knowledge hiding may, in turn, be related to turnover intentions. As Serenko and Bontis (2016) show, knowledge hiding will lower employees’ commitment to the organization, which will raise the probability of leaving the organization. Cerne and the collaborators (2014) show that knowledge hiding behaviors from one employee will lead to reciprocation from others, resulting in them being stuck in a knowledge hiding loop characterized by mistrust and negative feelings. Such a negative climate will lead to a higher chance of employee turnover [41]. When individuals perceive the organization as being supportive, the chances of knowledge hiding will be lower and, as such, the turnover intentions will have a lower probability.

Therefore, we propose the following hypothesis:

**Hypothesis** **3** **(H3).** *Knowledge hiding mediates the relationship between perceived organizational support and turnover intentions*.

#### 1.1.5. Affective Commitment and Knowledge Hiding

Besides organizational factors that predict knowledge hiding, such as perceived injustice [42], diversity [10], organizational climate [16], or organizational culture, there are also personal factors that play an important role in the emergence of knowledge hiding, one of which could be the level of commitment displayed by an employee.

Organizational commitment can be defined as the strength of an individual’s identification with an organization and their level of involvement in the workplace [43]. Meyer and Allen (1991) [44] describe three components to commitment: affective commitment, continuance commitment, and normative commitment. Affective commitment is represented by an emotional bond with the organization, by identification with the organization and involvement in it. Employees with continuance commitment will remain with an organization based on how costly they perceive leaving the organization would be. Normative commitment implies staying in an organization based on a feeling of obligation to continue working there [44].

Out of the three forms of commitment, we expect that affective commitment may be related to knowledge hiding behaviors. Brown and his colleagues (2005) [45] propose the idea of territoriality, seen as the tendency of employees to behaviorally express feelings of ownership of a physical or social object. It is well documented that when individuals share feelings of territoriality towards the information they possess, it will lead to more knowledge hiding behaviors [5,7,46]. But when the employees manifest affective commitment based on an emotional attachment to the organization, they may not see the information as their possession, but as a common good in the organization [15].

In addition, an affective commitment based on emotional attachment for the organization will be a source of identification with the organization. According to the social identity theory [21], affect can lead to a process of social categorization, a process that is at the basis of forming the social identity. Based on positive feelings for the organization, it is probable that it will be a source of an employee’s identity. When identification occurs, the probability of taking on knowledge hiding behaviors is reduced because the individual’s goals are aligned with the organizational goals [22].

Therefore, we propose the following hypothesis:

**Hypothesis** **4** **(H4).** *Affective commitment is negatively associated with knowledge hiding*.

#### 1.1.6. Knowledge Hiding as a Mediator between Affective Commitment and Organizational Citizenship Behaviors

As previously argued, citizenship behaviors represent a vital component of an organization’s life [29,30,31]. One predictor for this kind of behavior may reside in the type of commitment displayed by an employee [47]. It seems that effective commitment has the strongest relationship with organizational citizenship behaviors, especially with altruism and compliance, compared to normative and continuance commitment [48]. We argue that effective commitment will lead to a higher number of citizenship behaviors these employees take an interest in promoting the organization’s well-being. Thus, employees that have high levels of affective commitment want to be part of the organization for a long time and employ extra effort to make sure that the organization is successful.

Also, the relationship between affective commitment and citizenship behaviors can be explained by the social exchange theory [27,49]. Given that the organization triggers good feelings in its employees, they will want to offer something back. One way to repay the organization is investing effort in citizenship behaviors.

A meta-analysis shows that affective commitment is negatively related to an external locus of control [48]. Thus, individuals that present affective commitment see themselves as responsible for work-related outcomes. This may be a sufficient motivator for them to not keep the information they possess for themselves in order to promote organizational performance.

Drawing on these assumptions, we expect that the direct relationship between organizational affective commitment and organizational citizenship behaviors may be explained by knowledge hiding. Given that organizational affective commitment will lead to employees not being territorial [45] to the information they own, they may be motivated to display more organizational citizenship behaviors oriented toward helping others. In addition, the affective commitment will lead to an overlap between employees’ goals and the organization’s goals [22] which will lower the probability of engaging in knowledge hiding, which, in turn, will lead to more citizenship behaviors oriented towards common goals.

Therefore, we propose the following hypothesis:

**Hypothesis** **5** **(H5).** *Knowledge hiding mediates the positive relationship between affective commitment and organizational citizenship behaviours*.

#### 1.1.7. Knowledge Hiding as a Mediator between Affective Commitment and Turnover

Affective commitment characterized by positive emotions towards the organization [47] may play an important role in employees’ decisions about leaving the organization [50,51]. Whitener and Walz (1993) [52] show that, based on a social exchange view [27], the presence of affective commitment, not other kinds of commitment, leads to lower levels of turnover because employees feel the need to give back to the organization that provides a good environment. Meyer and the collaborators (1990) [53] show that emotional commitment represents a state that binds employees to the organizations.

Consequently, knowledge hiding may explain the relationship between affective commitment and turnover intentions. Knowledge hiding will be associated with an unhealthy unethical environment that employees will be motivated to leave [15]. If individuals are affectively committed to the organization, lowering knowledge hiding behaviors will reduce the perception of the environment as being unhealthy, so that employees will not manifest turnover intentions.

Thus, we propose the following hypothesis:

**Hypothesis** **6** **(H6).** *Knowledge hiding mediates the negative relationship between affective commitment and turnover intentions*.

## 2. Method

### 2.1. Sample and Procedure

The study was conducted in the ambulance setting from two different counties in an East-European country using a convenience sampling method. Five hundred and forty-five questionnaires were handed out, of which 324 were filled out and returned. The valid sample consisted of 305 participants working as medical or paramedical professionals providing emergency medical support to people who are injured or critically ill and transporting them to a medical facility, if necessary. Most ambulance journeys could be nonemergency but vital to patients. The ultimate task of the ambulance staff is to assess the medical needs of the sick or injured. Following the assessment, they provide the medical assistance needed. This means highly trained professionals carrying out life-saving procedures, making decisions on treatment, and administering it on the spot, sometimes under difficult conditions. In so doing they share information with each other and collaborate to deliver the very best care for the patients. In between responding to emergencies, the professionals working in the ambulance setting file reports and fill out forms related to the calls they have been on. Also, ambulance staff needs to communicate with the hospital staff about the patient’s condition keeping track of the procedures they have performed and medications they have administered. They usually work on teams and are required to communicate and coordinate their activities closely to face stressful situations. In terms of work schedule, the participants worked full time, in shifts. The shifts could run from 8 to 12 h (with a couple of days off after), but their rotations are rarely the same from week-to-week. Because they must be available to work in emergencies, they may work overnight and on weekends.

Prior to data collection, the management of the two organizations has presented the goals and the procedure of the study, and the informed consent was obtained in line with the local ERB regulations. Paper-and-pencil surveys were completed on-site during regular work hours and returned in sealed envelopes. To reduce social desirability bias and to protect the rights of our participants, the questionnaire was accompanied by a cover letter in which anonymity, confidentiality, and the voluntary nature of participation were emphasized.

The average age of the respondents was 43.23 (SD = 8.84) and 62.5% were male. The average tenure in the organization was 13.58 years (SD = 8.39). The respondents belonged to various hierarchical and functional levels: doctors (4.5%), nurses (46.2%), and ambulance drivers (49.3%).

### 2.2. Measures

Participants reported on perceived organizational support, affective commitment, knowledge hiding, organizational citizenship behaviors, and turnover intentions. We also obtained demographic information. Given the fact that the variables are, in general, at the level of psychological perception, the self-reported measurement method is appropriate. All measures originally developed in English were translated into the local language, and back-translated using the procedures recommended by the International Test Commission Guidelines (2005). Unless otherwise noted, all items were assessed on a 7-point Likert-type scale on which 1 = “not at all” and 7 = “to a very great extent”. Scale items were averaged such that higher scores indicated a greater degree of the underlying construct.

Perceived organizational support. Following the theoretical conceptualization based on organizational support theory, we estimated perceived organizational support as a generalized perception concerning the extent to which the organization values the employees’ contributions and cares about their well-being [20]. To assess perceived organizational support, we used a 16-item scale developed by Eisenberger et al., 1986. Sample items are, “The organization strongly considers my goals and values” and “The organization fails to appreciate any extra effort from me” (α = 0.90).

Affective commitment. The Affective subscale of the Organizational Commitment Questionnaire [54] was administered. Its items (e.g., “I really feel as if this organization’s problems are my own”) assess employees’ affective bond to their organization, in terms of willingness to remain in their organization because they want to [55]. Items have been rated on a 5-point scale ranging from 1 (“strongly disagree”) to 5 (“strongly agree”). The Cronbach’s alpha was 0.64.

Knowledge hiding. Knowledge hiding was assessed with a 12-item scale developed by Connelly (2012) [12]. The scale was preceded by the following statement: “In a specific episode in which a particular co-worker requested knowledge from you and you declined.” It further includes items such as “I pretended I did not know what s/he was talking about.” Hereby, the items assess the intentional attempt to withhold or conceal knowledge that has been requested by another individual. The three sub-dimensions of knowledge hiding were: evasive hiding, playing dumb, and rationalized hiding. Evasive hiding was measured with four items and the internal consistency was good (α = 0.86). Playing dumb was also measured with four items, including “said I didn’t know even though I did” and demonstrated good internal consistency (α = 0.88). Rationalized hiding was measured with four items, as well, including “explained that I would like to tell him/her but was not supposed to” and demonstrated good internal consistency (α = 0.93). It is important to note that the requests for knowledge came from individuals, not from groups or organizations [12]. The situations in which employees keep silent or keep secrets (as no knowledge has been requested), or the situations focusing on other levels of analysis, such as group (e.g., [56]), organizational (e.g., [57]), or inter-organizational (e.g., [58]) are not considered.

Organizational citizenship behaviors. Organizational citizenship behaviors consist of behaviors of a discretionary nature that are not part of employees’ formal role requirements, but nevertheless, promote the effective functioning of the organization [32]. The scale developed by Podsakoff and colleagues (1990) [59] has 24 items (e.g., “I help others who have heavy workloads”, “I consume a lot of time complaining about trivial matters”). The Cronbach’s alpha was 0.79.

Turnover intentions. Participants’ turnover intentions were measured with four modified items taken from the scale developed by Kelloway, Gottlieb, and Barham (1999) [60]. These items referred to participants’ intentions to quit their job. Sample item stated, “I intend to ask people about new job opportunities.” Participants rated items using the 5-point Likert scales that vary from strongly disagree (1) to strongly agree (5). The scale was internally consistent (α = 0.85).

Control variables. We collected data on a number of controls which, based upon prior literature were deemed possible variables which may account for turnover intentions and organizational citizenship behaviors. For example, longer-tenured employees generally have higher in-role performance and display more organizational citizenship behaviors. According to a meta-analysis, the age (rather than organizational tenure) was largely unrelated to core task performance but had stronger relationships with citizenship performance and counterproductive performance [61]. Tenure and age were reported in years.

We tested additional models with other control variables including participants’ age, gender, organizational tenure, all of which might affect the variables in the model. However, these variables did not alter the results substantially and are excluded here.

## 3. Results

Before starting with the actual model testing, we checked the potential influence of the control variables on the dependent variables. Age, gender, and professional level have been shown in other studies to influence turnover intentions or organizational citizenship behaviors. In our sample, there was no influence of gender on turnover intentions (t = 1.857, *p* = 0.064) or organizational citizenship behaviors (t = 1.378, *p* = 0.169). The average age of the participants was uncorrelated with turnover intentions (r = −0.031, *p* = 0.601) and organizational citizenship behaviors (r = −0.036, *p* = 0.556). As recommended by Becker [62] (2005), we excluded all non-significant control variables in subsequent analyses given that they were not significantly related to the dependent variables.

Table 1 presents the means, standard deviations, and bivariate correlations of all the variables in the study and demographics, as well. As can be seen, a strong negative relationship between perceived organizational support and knowledge hiding (r = −0.22, *p* < 0.0001) and between knowledge hiding and organizational citizenship behaviors (r = −0.29, *p* < 0.0001) were found. Moreover, knowledge hiding positively correlated with turnover intentions (r = 0.36, *p* < 0.0001) whereas affective commitment negatively correlates with knowledge hiding (r = −0.13, *p* < 0.05).

**Figure 1:**
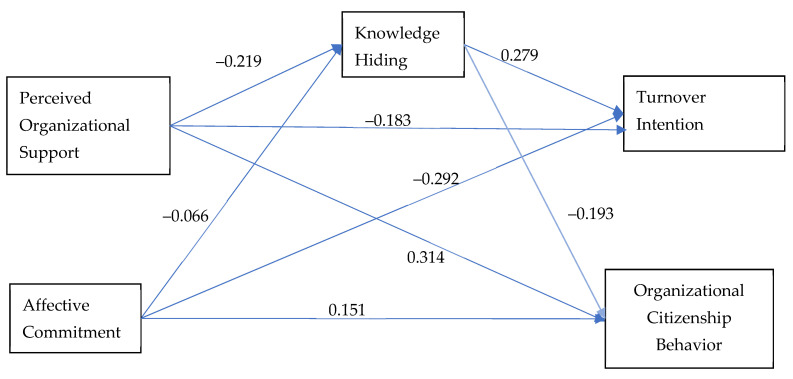
The research model.

All hypotheses were corroborated and a structural equation model (by IBM SPSS Amos 23. IBM Corp.: Armonk, NY, USA) was computed to simultaneously test all the relationships as predicted by the hypotheses (Figure 1).

Maximum likelihood estimation methods were used to test the different models. The goodness-of-fit of the models was evaluated using absolute and relative indices. The estimated measurement model showed a good fit to the data: χ^2^ = 0.146; *p* = 0.703; normed fit index (NFI) = 0.99, CFI = 1, and RMSEA = 0.00. Therefore, we used it to calculate the following structural models. Since there is no single statistical test that best describes the strength of a model’s predictions, several measures of approximation were employed. In the Normed Fit Index (NFI), the Goodness-of-Fit Index (GFI), and the Comparative Fit Index (CFI), a degree of fit above 0.9 is considered sufficient [63], whereas RMSEA (the root mean square error of approximation) with values of 0.06 or less indicates good model fit [64]. This allowed us to proceed in testing the hypotheses.

Specifically, Figure 1 indicates that perceived organizational support relates directly and negatively to knowledge hiding (β = −0.219, *p* < 0.0001) and positively to organizational citizenship behaviors (β = 0.314, *p* < 0.0001), whereas knowledge hiding relates negatively to organizational citizenship behavior (β = −0.193, *p* < 0.0001). Knowledge hiding is positively associated with turnover intentions (β = 0.279, *p* < 0.0001) and turnover intentions is negatively associated with perceived organizational support (β = −0.183, *p* < 0.0001). Effective commitment relates directly and negatively to turnover intentions (β = −0.292, *p* < 0.0001), but not to knowledge hiding (β = −0.066, *p* > 0.05).

In order to investigate the role of knowledge hiding in the relationships between perceived organizational support and the dependent variables—organizational citizenship behaviors and turnover intentions—we tested the indirect effects using the bootstrapping procedure described by Hayes (2013) [65] as well as Hayes’s PROCESS procedure for SPSS. Similarly, the indirect effects were reported for the relationships between affective commitment and the dependent variables. Effects are considered significant when the bootstrapped 95% CI around the indirect effect does not include zero.

Hypothesis 1 predicted that perceived organizational support would negatively affect knowledge hiding. As can be seen in Table 2, from the full-mediation model, the direct paths from perceived organizational support to knowledge hiding (β = −0.232, *p* < 0.001) were significantly negative.

The second hypothesis suggested that knowledge hiding would mediate the relationship between perceived organizational support and organizational citizenship behaviors. According to the mediation analysis, knowledge hiding is a significant mediator given that the indirect effect of perceived organizational support on organizational citizenship behaviors is positive and significant (β = 0.03, *p* < 0.001, CI [01; 0.04)]. The indirect effect size was 0.10 (SE = 0.03; CI [0.04; 0.19]. After controlling for the mediator, the direct effect of perceived organizational support on organizational citizenship behaviors remains significant (β = 0.205, *p* < 0.001). Although the indirect effect size is small, knowledge hiding partially mediates the effect of perceived organizational support on organizational citizenship behaviors, and Hypothesis 2 was supported by the data.

Hypothesis 3 predicted that knowledge hiding would mediate the effects of perceived organizational support on turnover intentions. The results supported the significance of the indirect effect of perceived organizational support on turnover intentions, 95% CI [−0.039, −0.003], through knowledge hiding (β = −0.02, *p* < 0.001). The indirect effect size was 0.09 (SE = 0.06; CI [0.01; 0.25]. The direct effect of perceived organizational support on turnover intentions is negative and significant (β = −0.194, *p* < 0.001). Therefore, with a small indirect effect size, knowledge hiding partially mediates the effect of perceived organizational support on turnover intentions.

The fourth hypothesis stated that affective commitment is negatively associated with knowledge hiding. According to the mediation model, the direct paths from affective commitment to knowledge hiding (β = −0.219, *p* < 0.05) were significantly negative.

The fifth hypothesis predicted that knowledge hiding mediates the positive relationship between affective commitment and organizational citizenship behaviors. In order to test the mentioned relationships, we employed the bootstrapping mediation analysis proposed by Preacher and Hayes (2013) [66]. The results supported the significance of the indirect effect of affective commitment on organizational citizenship behaviors, 95% CI [−0.197, −0.080], through knowledge hiding (β = −0.138, *p* < 0.001). The indirect effect size was 0.03 (SE = 0.014; CI [0.004; 0.061]. The direct effect of affective commitment on organizational citizenship behaviors is positive and significant (β = 0.208, *p* < 0.001, CI [0.111; 0.304]. Based on these results, knowledge hiding partially mediates the effect of affective commitment on organizational citizenship behaviors.

The last hypothesis stated that knowledge hiding would mediate the relationship between affective commitment and turnover intentions. According to the mediation analysis, knowledge hiding is a significant mediator given that the indirect effect of affective commitment on turnover intentions is negative and significant. The indirect effect was −0.050 (CI [−0.124; 0.004]). After controlling for the mediator, the direct effect of affective commitment on turnover intentions remains significant (β = −0.418, *p* < 0.001). Thus, the last hypothesis was supported.

## 4. Discussion

The core aim of this paper was to disentangle how the perceived organizational support and emotional commitment are linked to organizational citizenship behaviors and turnover intentions by the means of knowledge hiding as an explanatory mechanism.

Building on social identity theory [21] and the social exchange theory [27], we argued that high levels of perceived organizational support will lead to lower levels of knowledge hiding. The results supported this hypothesis. In other words, when employees face an environment perceived as supportive, they will not be tempted to hide information because they identify more with the organization [22], and they feel like they should give something back to the organization [28]. This comes in line with other studies from the literature that document the importance of creating a positive relationship between employees and their organizations [67]. Moreover, studies show that abusive leadership promotes knowledge hiding [67,68], underlining the importance of showing care and offering support for the employees.

Next, we tested two mediation hypotheses aiming at explaining the way perceived organizational support predicts organizational citizenship behaviors [31,34,35] and turnover intentions [15,41], with knowledge hiding acting as an explanatory mechanism. The mediation analyses support these two hypotheses. When employees benefit from perceived organizational support, their knowledge hiding behaviors are reduced. The reduction of knowledge hiding behaviors is what explains the positive effect of perceived organizational support of organizational citizenship behaviors and the negative effect on turnover intentions. These results may underline the importance of receiving support from the organization—employees do not perceive that they need to protect themselves by hiding knowledge, which will be beneficial to both individuals and the organization.

One interesting finding represents the significant indirect effect of perceived organizational support and citizenship behaviors via knowledge hiding. Other studies did not find a significant mediation [67], while our study did. It is possible that not only culture is important [67], but also the job’s profile, as the medical domain may involve high levels of complexity and urgency, alongside a more individualistic approach to compensation.

Building on territoriality [5,7,46] and social identification [21], the third hypothesis stated that emotional commitment to the organization is linked to knowledge hiding behaviors in the organization. The results support this statement. As such, when individuals are emotionally attached to their organizations, they will be less likely to engage in knowledge hiding behaviors. These results come in line with what Jahanzeb and the collaborators (2020) [69] showed—disidentification from one’s organizational context promotes knowledge hiding. Thus, it becomes clear that knowledge hiding is strongly influenced by work attitudes displayed by employees.

The last two hypotheses implied another two mediation analyses, one for the link between affective commitment and organizational citizenship behaviors [45,48,49] and one between affective commitment and turnover intentions [51,52,53] both by the means of knowledge hiding. The mediation analyses support these two hypotheses. Thus, when employees’ affective commitment to their organization is high, their knowledge hiding behaviors will be reduced. The reduction of knowledge hiding behaviors is what explains the positive effect of the affective commitment on organizational citizenship behaviors and the negative effect on turnover intentions. These results are aligned with the idea that the positive feelings triggered by the organization play an important role in the approach the individual has towards the organization [27,49].

### 4.1. Implications

The present study has several implications, both at a theoretical level and a practical one. At the theoretical level, the study adds to the literature on knowledge hiding by identifying complex dynamics in which it is involved. On one hand, we show that perceived organizational support and affective commitment towards the organization are important predictors for knowledge hiding, adding to the literature regarding the antecedents of knowledge hiding. As previous studies focused on organizational elements that promote the emergence of knowledge hiding [70,71], we show the importance of factors that may inhibit it, prompting a positive approach and alternatives in organizations. We thus integrate the literature of elements that should be avoided in the context of an organization with the one focused on appliable, positive alternatives [71,72].

Second, we add to the literature on knowledge hiding and organizational outcomes by underlining the important indirect role knowledge hiding plays regarding both positive consequences (citizenship behaviors) and negative consequences (turnover intentions). It is important that our study looks into employee behaviors that indirectly affect organizational performance. Organizations that promote care towards their employees and manage to construct affective commitment will be subject to less information hiding. This will be beneficial for promoting positive extra-role behaviors and preventing employees from leaving the organization. Studying potential mechanisms that explain organizational outcomes is important as it helps us disentangle the complexities of organizational dynamics and pinpoint elements open for interventions.

On a practical level, the study answers the call of Anand and the collaborators [70] regarding directions for HR practitioners. In order to lessen the negative effect of knowledge hiding, our study offers guidelines for developing strategies and interventions aimed at creating a supportive organizational environment and raising the affective level of employees. First, our recommendations are directed towards creating a supportive organizational climate. Thus, organizations that show their employees that they are valued and are oriented towards showing care to their employees may be protected from the emergence of knowledge hiding. Based on the obtained results, organizations should make sure that their employees’ needs are fulfilled, while also showing appreciation for their efforts and work. Noticing and praising employees will, thus, protect the organization against knowledge hiding behaviors.

Second, our practical recommendations are oriented towards ensuring the commitment of employees, especially affective commitment. In this case, practicians should make sure that their employees really do feel like part of the organization, identifying with the organization and its needs. As results show, encouraging affective commitment in the organization, employees will refrain from knowledge hiding, leading to beneficial outcomes in the organization.

Moreover, the previous recommendation may aim not only at reducing knowledge hiding, but also prompting knowledge sharing. Raising the levels of knowledge sharing in an organization will enable the stakeholders and team to harvest multiple benefits, such as innovation [73] or task performance [74,75]. Thus, given that work teams are more and more diverse, knowledge sharing may offer a way to integrate goals and positive social interaction towards higher performance both in intra-organizational settings [74] and inter-organizational settings [76].

### 4.2. Limitations and Suggestions for Future Research

The study has several limitations. First, we have to take into consideration the fact that knowledge hiding is under-reported and a low-base-rate event because it is seen as an undesirable work-related behavior [12]. Even if the self-reported data may not reflect reality, knowledge-hiding behaviors involve concealing information, so it cannot be externally assessed [12].

Second, the fact that the study is based on self-report could be a liability as it raises the problem of the common method variance. The hypothesis testing is based on single-source data, therefore, some additional analyses have been employed. First, self-reported data is most problematic for topics that generate strong feelings, such as attitudes [77]. Dimensions of behavior or performance might be a less emotionally laden subject, and hence the potential distortion by self-reports is reduced. In addition, the response range was diverse [78], so that the social desirability bias was not apparent in our sample.

Next, the study does not run an experimental design, thus any causal claims are not appropriate. Future studies could replicate our findings while using an experimental design in order to further establish the validity of our results.

We still have much to discover about knowledge hiding and the actions needed to discourage these behaviors in organizations. Future research should also look into the contagion of knowledge hiding—when employees perceive others as hiding information, they will be affected, and they will develop resistance. In addition, future studies should focus on a longitudinal design in order to explore contagion effects in a more valid way.

## 5. Conclusions

The main goal of this study was to look into the antecedents of knowledge hiding in organizations and knowledge hiding as an explanatory mechanism for the relationship between perceived organizational support and organizational citizenship behaviors and turnover intentions, respectively between affective commitment and organizational citizenship behaviors and turnover intentions. The results support perceived organizational support and affective commitment as antecedents of knowledge hiding. Both knowledge hiding as a mediator between perceived organizational support and organizational citizenship behaviors and turnover intentions and knowledge hiding as a mediator between affective commitment and organizational citizenship behaviors and turnover intentions were supported. As employees will remain unmotivated to share their knowledge and will sometimes intentionally withhold it, scholars need a new, deeper understanding of what triggers individual knowledge hiding, its negative effects on employees, and how it can be mitigated in organizations.

## Figures and Tables

**Table 1:** Descriptive Statistics and Intercorrelations among Variables.

Variable	*M*	*SD*	1	2	3	4	5	6	7
1. Age	43.23	8.34	-						
2. Work exp. ambulance	13.58	8.39	0.65 **	-					
3. Gender	1.38	0.48	−0.13 *	−0.03	-				
4. Perceived organizational support	5.29	0.96	0.03	0.04	0.09	-			
5. Affective commitment	4.01	0.64	0.06	0.00	0.03	0.28 **	-		
6. Knowledge hiding	1.91	1.04	0.05	−0.003	−0.07	−0.22 **	−0.13 *	-	
7. Organizational citizenship behaviours	5.82	0.57	−0.04	−0.05	0.09	0.40 **	0.26 **	−0.29 **	-
8. Turnover intentions	1.46	0.76	−0.03	−0.04	−0.11	−0.32 **	0.38 **	0.36 **	−0.25 **

Note. *n* = 305; Gender: 1 = male; 2 = female. * *p* < 0.05; ** *p* < 0.01. Work exp. Ambulance = experience with working on an ambulance.

**Table 2:** Regression analyses.

	Knowledge Hiding		Organizational Citizenship Behaviours		Turnover Intentions	
Antecedent	Coeff.	SE	Coeff.	SE	Coeff.	SE
Perceived organisational support	−0.232 ***	0.061	0.205 ***	0.032	−0.194 ***	0.041
Knowledge hiding			−0.104 ***	0.030	0.189 ***	0.039
Constant	3.121 ***	0.326	4.942 ***	0.192	2.107 ***	0.249
			R^2^ = 0.187 F(2, 288) = 33.225 ***		R^2^ = 0.165 F(2, 294) = 29.040 ***	
Affective commitment	−0.219 *	0.095	0.208 ***	0.049	−0.418 ***	0.061
Constant	2.787 ***	0.387	5.260 ***	0.214	2.700 ***	0.267
			R^2^ = 0.136 F(2, 288) = 22.751 ***		R^2^ = 0.248 F(2, 294) = 48.463 ***	

Note. Unstandardized regression coefficients are presented in the table * p < 0.05; *** p < 0.001.

## Data Availability

Data is available upon substantiated request from the corresponding author.

## STROBE Guidelines

We confirm that this study is STROBE compliant.
